# P-794. A Feedforward Neural Network Predictive Model of Asymptomatic Bacteriuria (ASB) vs. Urinary Tract Infections (UTIs) – A Proof-of-Concept Assessment

**DOI:** 10.1093/ofid/ofaf695.1004

**Published:** 2026-01-11

**Authors:** Revanth S Yendamuri, Fatima Abdulle, Jashanjit K Turka, Jeffrey Solomon, Ken Koon Wong, Shubhayu Bhattacharyay

**Affiliations:** Cleveland Clinic Foundation, Stow, OH; Cleveland Clinic, Akron, Ohio; Cleveland Clinic Akron General Hospital, Akron, Ohio; Cleveland Clinic Akron General, Akron, Ohio; Cleveland Clinic, Akron, Ohio; Harvard Medical School, Boston, Massachusetts

## Abstract

**Background:**

Urinary tract infections (UTIs) are a common cause of healthcare visits and hospitalizations. A recent study attributed about 8.7 million ER visits to UTIs from 2016 to 2023^1^. Asymptomatic bacteriuria (ASB) is often mistaken for a UTI, leading to unnecessary treatment and the development of resistant bacteria. This study aims to develop a deep learning model to differentiate ASB from a UTI based on urinalysis parameters and to compare its performance to traditional statistical methods, specifically logistic regression.

**Figure 1.**
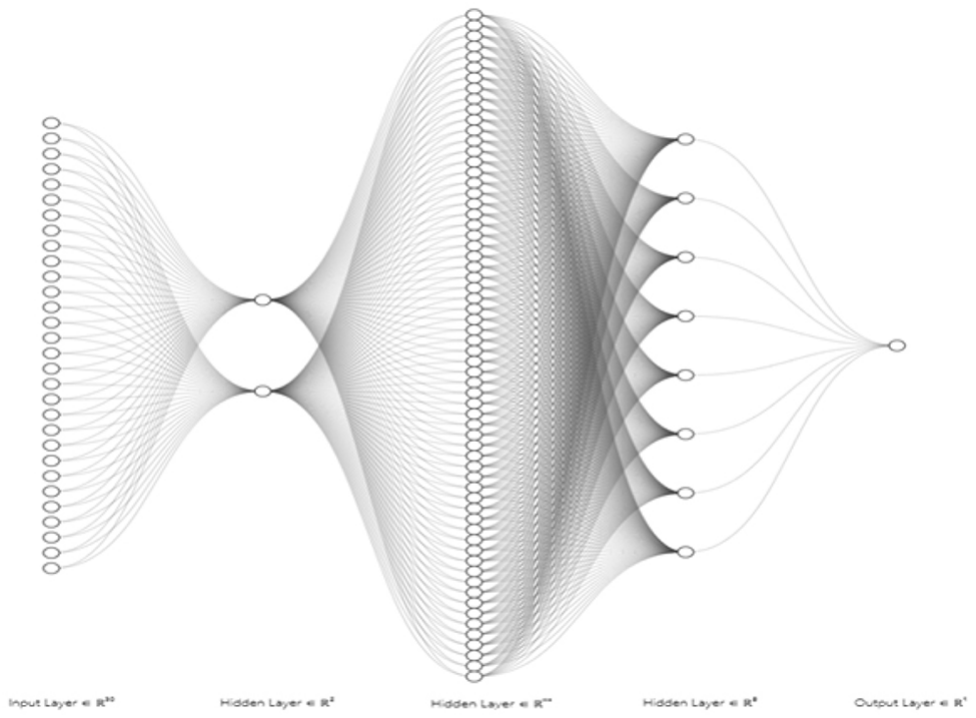
Feedforward neural network model with 3 hidden layers and 2, 128, and 8 nodes in their respective layers.

**Figure 2.**
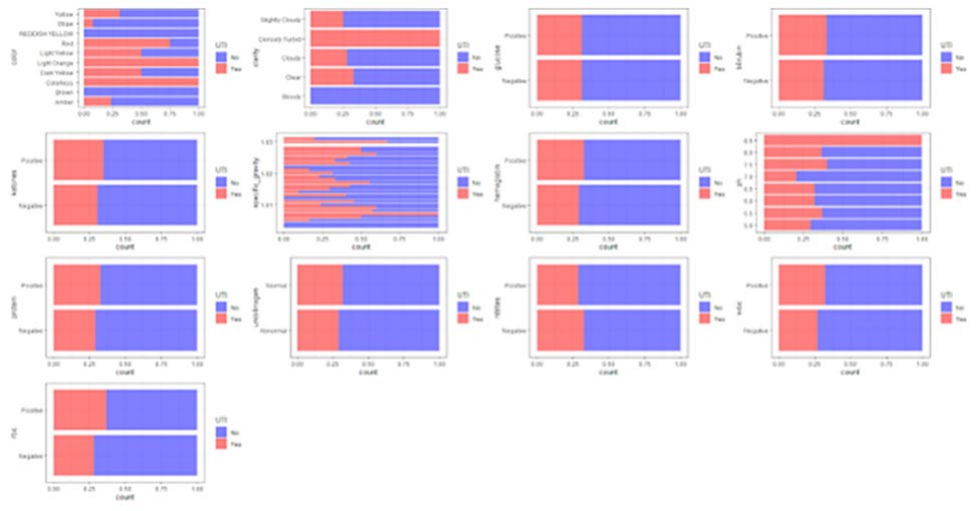
Proportion of ASB (Blue) vs. a UTI (Red) of urinalysis parameters.

**Methods:**

This retrospective case-control study included adult patients with ASB or UTIs from January 1^st^, 2010 to December 31^st^, 2022. Urinalysis parameters and urine culture results were collected. A feedforward neural network with 30 input features and 2 hidden layers was trained and tuned to predict either ASB or a UTI (Figure 1). The model's performance was compared to a stepwise bidirectional logistic regression model.

**Results:**

272 patient encounters were included (85 UTI, 187 ASB). The neural network model was found to be the optimal hyperparameter for the model based on the highest validation AUC of 0.69 (Figure 1). The tuned model achieved a binary accuracy of 0.83, precision of 0, and recall of 0 on the validation set with batch size of 4 and learning rate of 0.0003. The testing set showed an AUC of 0.35, binary accuracy of 0.842, precision of 0, and recall of 0 (Figure 2). The stepwise bidirectional logistic regression model, with an AIC of 270.65, demonstrated an accuracy of 0.745, but a precision of 0, a recall of 0, and an AUC of 0.525. Statistically significant parameters (p< 0.01) included red color (estimate: 0.6956), straw color (estimate: -0.292), and densely turbid clarity (estimate: 1.696).

**Conclusion:**

While the neural network model showed some predictive capabilities, the logistic regression model had higher accuracy. However, it also lacked precision and recall, indicating limitations in its ability to correctly identify UTI cases. Future tests with larger datasets may improve the models' accuracy in differentiating ASB from a UTI, if such patterns exist. Our results highlight the complexity of developing a robust predictive model for differentiating ASB vs. a UTI and the need for continued research on the topic.

**Disclosures:**

All Authors: No reported disclosures

